# Development and assessment of floor and ceiling items for the PROMIS physical function item bank

**DOI:** 10.1186/ar4327

**Published:** 2013-10-03

**Authors:** Bonnie Bruce, James Fries, Bharathi Lingala, Yusra Nazar Hussain, Eswar Krishnan

**Affiliations:** 1 Stanford University Department of Medicine, Division of Rheumatology & Immunology, 1000 Welch Rd., Suite 203, Palo Alto, CA 94304, USA; 2 Stanford University Department of Medicine, Division of General Internal Medicine, 211 Quarry Road, Suite 302, MC 5988, Palo Alto, CA 94304-1426, USA

## Abstract

**Introduction:**

Disability and Physical Function (PF) outcome assessment has had limited ability to measure functional status at the floor (very poor functional abilities) or the ceiling (very high functional abilities). We sought to identify, develop and evaluate new floor and ceiling items to enable broader and more precise assessment of PF outcomes for the NIH Patient-Reported-Outcomes Measurement Information System (PROMIS).

**Methods:**

We conducted two cross-sectional studies using NIH PROMIS item improvement protocols with expert review, participant survey and focus group methods. In Study 1, respondents with low PF abilities evaluated new floor items, and those with high PF abilities evaluated new ceiling items for clarity, importance and relevance. In Study 2, we compared difficulty ratings of new floor items by low functioning respondents and ceiling items by high functioning respondents to reference PROMIS PF-10 items. We used frequencies, percentages, means and standard deviations to analyze the data.

**Results:**

In Study 1, low (*n *= 84) and high (*n *= 90) functioning respondents were mostly White, women, 70 years old, with some college, and disability scores of 0.62 and 0.30. More than 90% of the 31 new floor and 31 new ceiling items were rated as clear, important and relevant, leaving 26 ceiling and 30 floor items for Study 2. Low (*n *= 246) and high (*n *= 637) functioning Study 2 respondents were mostly White, women, 70 years old, with some college, and Health Assessment Questionnaire (HAQ) scores of 1.62 and 0.003. Compared to difficulty ratings of reference items, ceiling items were rated to be 10% more to greater than 40% more difficult to do, and floor items were rated to be about 12% to nearly 90% less difficult to do.

**Conclusions:**

These new floor and ceiling items considerably extend the measurable range of physical function at either extreme. They will help improve instrument performance in populations with broad functional ranges and those concentrated at one or the other extreme ends of functioning. Optimal use of these new items will be assisted by computerized adaptive testing (CAT), reducing questionnaire burden and insuring item administration to appropriate individuals.

## Introduction

Physical Function (PF) is frequently measured using patient reported outcomes (PRO). The original 20-item ("Legacy") Health Assessment Questionnaire Disability Index (HAQ) [1,2] and the original 10-item ("Legacy") SF-36 Physical Function form (PF-10) [3] are widely used traditional PRO instruments. Both have demonstrated good reliability across diverse conditions and in different administration modes. However, they do not reliably measure physical function ability in the very able or the very disabled. They need new items at the extremes of function and populations at the extremes to validate the new items.

The Patient-Reported-Outcomes Measurement Information System (PROMIS), part of the NIH Roadmap Initiative, has developed improved tools for assessing PRO PF endpoints using Item Response Theory (IRT) methodology [4]. During its initial funding cycle, PROMIS developed a 154-item physical function core item bank. PROMIS chose the term "physical function" rather than "disability" to describe this domain to encourage development and use of items that measure functional status across the entire range of ability, from those who are physically strong and healthy to the least able-bodied.

The PROMIS PF core item bank, accessible from the PROMIS website [5], consists of 30 legacy items from the original HAQ and PF-10, which have been IRT-calibrated, and 124 new IRT-calibrated PROMIS items. In addition, a 10-item and a 20-item instrument, composed of items selected as the "best" from the PROMIS PF item bank [6] and Computerized Adaptive Testing (CAT) items, are accessible from the PROMIS website for investigational use.

The IRT-calibrated PROMIS physical function items have exhibited excellent performance and responsiveness in the populations studied, which contain few subjects at the floor or above the current ceiling [5]. Along with the new 10-item PROMIS PF-10 and the 20-item PROMIS PF-20 instruments, they achieve improvement in precision by use of improved items in better ways. Measurement precision is increased by about 50%, and substantial reductions in sample size requirements are achieved [5,7].

Despite these improvements, the current instruments are limited in their ability to measure the full spectrum of physical function. They lack items with a broad enough range to adequately measure the extremes. This means that persons at the floor, that is, institutionalized and severely or completely disabled persons, and those at the ceiling, that is, individuals with no functional impairment or those who have abilities far greater than average, are not appropriately assessed.

Floor and ceiling effects negatively affect measurement properties, including sample size requirements. When the functional ability range of a study population does not match assessment ability of the study, for example, there are insufficient items to capture the full range of participant functional ability, the need for larger sample sizes is increased. For example, Table 1 shows a comparative measurement range among five physical function instruments. The legacy PF-10 covers a range of only 2.3 standard deviations, the PROMIS PF-10 covers 3.1 standard deviations, the legacy HAQ covers 4.1 standard deviations, the PROMIS PF-20 covers 4.8 standard deviations, and the PROMIS 10-item Computerized Adaptive Testing (CAT) application, covers 5.7 standard deviations [7]. Reducing floor and ceiling effects can improve study efficiency by decreasing the sample size required for detecting group differences, in both randomized controlled trials and observational studies [7].

**Table 1:** Comparative measurement range of five physical function instruments with 90% reliability

	PF-10	HAQ	PROMIS PF-10	PROMIS PF-20	PROMIS PF CAT-10
Floor	-2.5	-4.5	-2.9	-4.5	-4.5
Ceiling	-0.2	-0.4	0.2	0.2	1.2
Range	2.3	4.1	3.1	4.8	5.7

Each unit below zero (the population mean) is scored as a negative of standard deviations toward the floor, and each unit above zero (the population mean) is a standard deviation in the direction of the ceiling "range" as the algebraic sum of standard deviations of positive and negative [15]. ^1 ^Instruments: PF-10 - 10-item physical function scale of the SF-36 [3]; HAQ - 20-item Health Assessment Questionnaire [2]; PROMIS PF-10 - PROMIS PF-10, 10-item Physical Function assessment form [5]; PROMIS PF-20 - PROMIS PF-20, 20-item physical function assessment form [5]; PROMIS PF CAT-10 - PROMIS PF 10-item Computerized Assessment Testing [5]

We sought to identify extant floor and ceiling items, create new items to close remaining gaps, and evaluate candidate items that could assess functional abilities of individuals at the lowest and highest levels of function. We set an *a priori *goal of an expanded PROMIS PF core item bank with 200 to 225 total items, including approximately 25 new floor and 25 new ceiling items. These numbers would be sufficient to create short forms for specific ranges of difficulty and would populate the extremes with approximately as many items per standard deviation as in the current core item set. This paper presents the two item-level evaluation studies describing the development of floor and ceiling items for the PROMIS physical function item bank and the comparison of difficulty ratings for the new items compared to items from the current core.

## Materials and methods

### Design

We conducted two cross-sectional studies. We sought to have new ceiling items asking about activities which were more difficult to do (requiring greater ability) compared to items in the PROMIS PF-10 and new floor items that involved activities that were less difficult (or easier) to do compared to items in the PROMIS PF-10. The objective of the first study was to identify extant potentially relevant items, construct additional candidate ceiling and floor items to fill in gaps, and evaluate the newly proposed items for clarity, importance and relevance. We adopted the PROMIS step-wise item development and evaluation protocols to identify, select and revise items and to assess item strengths and difficulties. The PROMIS network protocols describe standard methods for identifying, classifying and evaluating new items, as well as employing expert reviews and participant input using survey and focus group methods [5,8]. The objective of Study 2 was to compare item difficulty ratings for new floor and ceiling items, which were retained from Study 1, with items from the PROMIS PF-10. The studies were approved by Stanford University's Human Subjects Research Protection Program, and each participant gave written informed consent.

### Participant pool

To assess the new items we required a diverse participant pool that would include relevant subpopulations at the floor and at the ceiling. We sought to include arthritis patients, aging populations in their 80s and 90s, and nursing home residents, as well as able-bodied, physically fit athletes.

The initial pool of potential participants for the two studies consisted of 2,723 adult men and women. They included participants in our prior cohort studies (*n *= 2,490) from the Arthritis, Rheumatism, and Aging Medical Information System (ARAMIS) [9]. ARAMIS participants were composed of rheumatoid arthritis and osteoarthritis populations (*n *= 1,496) and healthy aging populations from the University of Pennsylvania longitudinal studies of aging (*n *= 325) [10] and the aging, osteoarthritis and exercise study (*n *= 669) [11]. We also added 30 additional participants from a local nursing home for floor item assessment, and 203 participants from a club of ultra-marathoners for ceiling item assessment.

### Participant classification for item evaluation

We classified participants to evaluate either floor or ceiling items based on disability scores. ARAMIS participant classification was based on scores from the original ("Legacy") HAQ [12]. The HAQ is composed of 20 items and is scored 0 to 3 with 3 being completely disabled. A HAQ score of 0.025 indicates little to no disability and a high functional ability. Disability scores of 0.75 to 1.0 indicate moderate disability and are commonly reported among subjects with rheumatoid arthritis or osteoarthritis [1,13]. The newly recruited ultra-marathoners and nursing home participants had no prior HAQ scores but were selected because of the high likelihood that most ultra-marathoners would be at or near the ceiling and nursing home residents would be at or near the floor.

**Figure 1:**
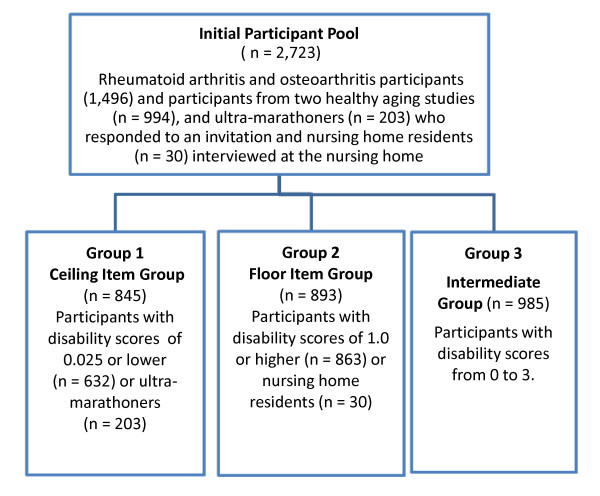
**Participant classification schema for the two study groups for evaluation of ceiling and floor items**.

Figure 1 shows how we identified participants for placement into one of three groups. Participants in Group 1 (*n *= 845), for assessment of ceiling items, had disability scores of 0.025 or lower or were members of the ultra-marathoner group. Group 2 participants (*n *= 893), for assessment of floor items, had disability scores of 1.0 or higher or were nursing home residents. Group 3 participants (*n *= 985), the intermediate group, had disability scores from 0 to 3. We composed our groups to permit comparison of ceiling items in ceiling and intermediate respondents and floor items in floor and intermediate respondents. The sample sizes for these studies were comparable to prior studies evaluating the current core physical function items for the PROMIS PF Item Bank [5,14].

### Item identification, selection and revision

We conducted extensive searches to identify extant items that assessed functional status at the extremes of ability. We searched the published literature for items in English language articles. We searched for candidate floor items that measured very easy and basic activities relevant to individuals with substantial and major impairments and for candidate ceiling items that measured difficult or challenging activities that would be appropriate for individuals with high functional ability and little to no functional impairment. We made no initial judgments about the quality of any item and selected those relevant to assessment of the floor or the ceiling.

We searched the Internet, including PubMed and Google Scholar for original articles and literature reviews. We used broad search terms to identify English language self-report instruments to insure capture of items that would assess some aspect of severely limited function and excellent or superior function. Our search terms included functional ability, disability, frailty, elderly, limitations, floor items, ceiling items, runners, mobility, physical activity, fitness, rehabilitation, independence measure, self-report and self-assessment questionnaires. Most publications discussed items only as part of the instruments and few included function items directed at the extremes.

Retained items were edited, wherever possible, to conform to the PROMIS conventions [8] of using the present tense, a capability stem ("Are you able", "Does your health now limit you"), no time frame, no disease attribution, and a five-item response set with the most negative response at the far right of the scale. For the "Are you able" stem, response options were "Without Any Difficulty", "With a Little Difficulty", "With Some Difficulty", "With Much Difficulty", and "Unable to Do". For the "Does your health now limit you" stem, response options were "Not at All", "Very Little", "Somewhat", "Quite A Lot", and "Cannot Do". The study investigators (BB, JFF, EK) conducted the initial item identification, selection and editing activities.

### Study 1: Evaluation of clarity, importance and relevance

**Figure 2:**
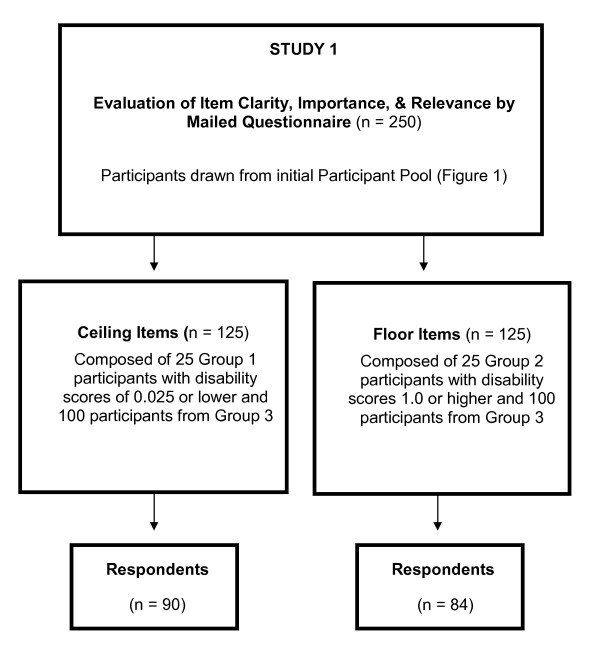
**Study 1 group formation and response rates**.

We sought to qualitatively evaluate the clarity, importance and relevance of new candidate items with participants who were representative of ceiling items and those who were representative of floor items. We invited 250 individuals from the initial participant pool to comprise two groups of 125 each to complete a mailed questionnaire (Figure 2). To assess ceiling items, we invited 25 participants from Group 1 and 100 participants from Group 3. To assess floor items, we invited 25 participants from Group 2 and 100 participants from Group 3. Participants were not compensated for questionnaire completion.

Participants were asked to rate each item's clarity ("Totally Clear", "Somewhat Clear", "Not Clear") and importance ("Very Important", "Somewhat Important", "Not important"). We asked them to provide feedback about item relevance and relationship to their activities of daily living. In addition, we conducted an audio-taped focus group to elicit feedback on floor items at a local nursing home. Because of the wide geographical distribution of ceiling participants, it was not feasible to conduct a focus group.

### Study 2: Comparison of item difficulty

**Figure 3:**
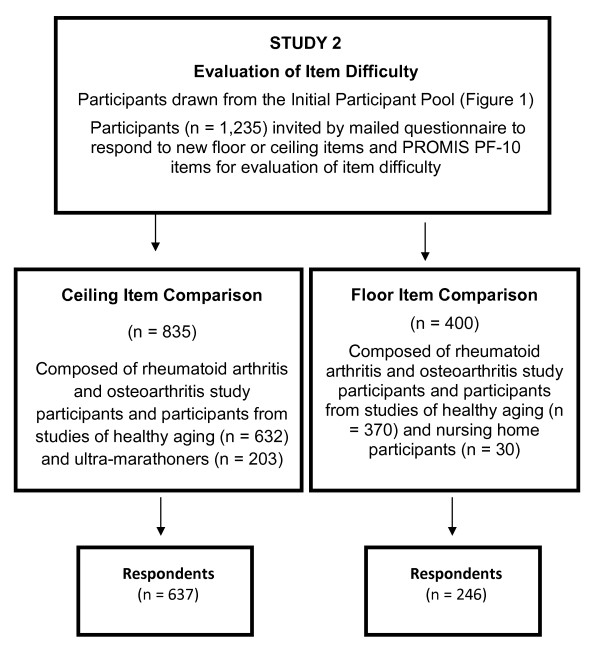
**Study 2 Group formation and response rates**.

Figure 3 shows how we selected participants for comparison of difficulty ratings of new floor and ceiling items relative to items in the PROMIS PF-10. We invited 1,235 participants from the initial participant pool to complete a mailed questionnaire that included the 10 PROMIS PF-10 items and either candidate floor or candidate ceiling items retained from Study 1. Ceiling item invitees consisted of members of the ultra-marathoner's club (*n *= 203) and rheumatoid arthritis and osteoarthritis study participants and participants from studies of healthy aging (*n *= 632), who had been selected for having a zero disability score. Floor item invitees consisted of nursing home residents (*n *= 30) and rheumatoid arthritis and osteoarthritis study participants and participants from studies of healthy aging (*n *= 370).

We combined the response options "With Much Difficulty" and "Unable to Do" to use as an indicator of an item's difficulty and ordered the percent of responses from highest (most difficult) to lowest (less difficult). To standardize the comparison for items with unique contexts and response options (such as asking how many minutes it takes to walk a mile) the most negative response option was considered equivalent to "With Much Difficulty" or "Unable to Do".

### Analyses

We used frequencies, percentages, means and standard deviations to describe the study groups. Participant assessment of clarity and importance was analyzed using percentages. Analyses were conducted with SAS 9.1 (SAS Institute, Cary, NC, USA).

## Results and discussion

### Participants

The initial participant pool (*n *= 2,723) was composed of 2,723 men and women, who were representative of the floor and ceiling. They were predominantly White, had 16 years of education, and averaged 72 years of age, ranging from 22 to 99 years. They had a wide range of functional ability, ranging from 0 to 3. Half of the participants with intermediate scores were women. Sixty-two percent of participants with disability scores lower than 0.025 were men. Participants with scores higher than 1.0 were the oldest (81 years). Those with disability scores lower than 0.025 were the youngest (61 years), and those with intermediate scores were in-between (72 years).

### Item identification, classification, selection and revision

We identified 165 published instruments covering diverse fields, including Rheumatology, Neurology, and Physical Medicine and Rehabilitation. Fifteen (9%) of the instruments contained at least one item assessing very easy abilities (at the floor), 13 (8%) contained at least one challenging or difficult item (at the ceiling), and 21 (13%) contained items assessing both extremes. Remaining instruments measured activities that were unrelated to assessment of the floor or ceiling of physical function (that is, they addressed pain, fatigue, satisfaction or were unrelated to the PROMIS definition of physical function).

We constructed candidate items to conform to the PROMIS format [14]. We maintained an item's context (for example, turning over in bed, running five miles) and revised the item's reference to the present time. We add a "capability" stem ("Are you able" or "Does your health now limit you") and applied the five standard PROMIS PF item response option set with the most negative response at the far right of the scale, with the exception of two unique floor items that accommodated only four response options. For the "Are you able ..." stem, response options were "Without Any Difficulty", "With a Little Difficulty", "With Some Difficulty", "With Much Difficulty", and "Unable to Do". For the "Does your health now limit you" stem, response options were "Not at All", "Very Little", "Somewhat", "Quite A Lot" and "Cannot Do".

To ensure that the item pool would include an array of relevant experiences at the extremes, we included items with unique contexts associated with common, recognized or widely understood activities. Such items included an assessment of speed and performance history: for example, "What is your best time for running one mile now?" or "Are you able to dress and groom yourself as quickly as you did five years ago?" Some items also had unique response options. For example, for a floor item: "In the past year, how much weight have you lost unintentionally?", the responses were: "None", "Up to 5 pounds", "5 to 10 pounds", and "11 pounds or more". For a ceiling item: "How many minutes does it take you to walk one mile, the responses were: "14 minutes or less?", "15 to 19 minutes", "20 to 24 minutes", "25 or more minutes", and "can't do".

All of the new candidate items were reviewed internally and sent by email for external review by PROMIS investigators using a modified Delphi approach over several iterations until no additional modifications were suggested. The remaining pool of 31 candidate floor and 31 candidate ceiling items then underwent evaluation in Study 1.

### Study 1: Evaluation of item clarity, importance and relevance

#### Floor items

##### Respondents

We had a 67% (*n *= 84/125) response rate to our mailed questionnaire for floor item evaluation (Figure 2), resulting from consenting new participants, re-consenting other participants and questionnaire completion. Sixty-four percent were White, and 59% were men. They were nearly 79 years old, had about 15 years of education, and a mean disability score of 0.62. Visual examination of the data suggested that there were no obvious response biases, for example, none of the respondents had marked the same response for all items.

##### Clarity, importance, and relevance ratings

The great majority (85% to 100%) of the items were rated "Totally Clear". Twenty-six (86%) of the floor items were rated "Totally Clear" by at least 95% of respondents. The five items rated "Totally Clear" by less than 95% of respondents were: "Hold a card/letter in order to read it"; "Move about your residence"; "Do you feel exhausted"; "Move about a dark room/hallway without falling"; and "Move from street to sidewalk without a curb cut".

The proportion of respondents rating items "Very Important" ranged from 35% to 83%. 83% (*n *= 26) of the items were considered "Very Important" by at least one-half of respondents. The two items rated as "Very Important" by the highest proportion of respondents (83%) were: "In the past year, how many times did you fall"; and the item measuring ability to move about their residence. The item with the lowest "Very Important" rating (35%), but the highest "Somewhat Important" rating (44%), was "Squeeze another person's hand". "Not Important" ratings were generally low, ranging from zero to 22%, indicating that most items were considered relatively important by the great majority of respondents. The item with the highest proportion (22%) of "Not Important" ratings was "Dress yourself in less than 10 minutes", followed by 21% of respondents rating "Not Important" the ability to "Loosen a screw using a manual screwdriver".

##### Respondent feedback

The majority of comments related to a respondent's own capability or interest in performing the activity or personal reasons for the item's insignificance. Item-related comments were largely related to requests for greater detail or specification about what was being asked.

#### Focus group

Seven White nursing home residents, all women, and ranging in age from 86 to 94 years old participated in the focus group. All participants were alert, able to interact, and responsive. Focus group participants rated most items as clear and important. When asked if there were any areas of physical function or related issues that were missing and which should be included for measuring activities of daily living in older adults, they offered no additional feedback.

#### Final floor item pool

The final floor item pool contained 30 of the original 31 items. Based on overall evaluation activities, we removed the item, "Dial a telephone number on the keypad of a cell phone". Twenty-six (84%) of the floor items had "Are you able to..." stems. The remaining four items had unique contexts and response option sets, asking about performance speed and distance, exhaustion, falls and unintentional weight loss.

#### Ceiling items

##### Respondents

We had a 72% (*n *= 90/125) response rate to our mailed questionnaire for assessment of candidate ceiling items, resulting from consenting new participants, re-consenting other participants and questionnaire completion. Respondents were 40% White, 36% men, averaged 62 years old, had about 15 years of education, and a mean disability score of 0.30. Visual examination of the data revealed no obvious response patterns or biases, for example, none of the respondents had marked the same response for all items.

##### Clarity, importance and relevance ratings

The proportion of "Totally Clear" ratings ranged from 78% to 100%. Twenty-five (81%) of the items were rated "Totally Clear" by at least 90% of respondents, indicating that most respondents found the items easy to understand. The proportion of respondents rating items as "Not Clear" ranged from zero to 6% across items, also suggesting that items were clear. The item evaluated as "Not Clear" by the highest proportion of respondents (6%) was "Painting a room". Respondent comments indicated that lack of detail interfered with clarity. For example, there were issues raised about the size of the room; number of paint colors, presence of molding or baseboard; use of a brush or roller; and need to paint the wall, ceiling and/or trim.

The proportion of respondents rating specific items as "Very Important" varied widely, ranging from 11% to 77%. The four items with the highest "Very Important" ratings (73% to 77%) were: "Transfer clothes from a washer to a dryer" (77%); "Total number of times doing physical activity that made them breathe hard" (74%); "Total time doing vigorous physical activity" (74%); and "Taking a 20-minute brisk walk without stopping to rest". The item rated Very Important by the lowest proportion of respondents (11%) was "Row a rowboat".

The proportion of respondents rating Items "Not Important" also varied widely, ranging from 6% to 73%. The item rated "Not Important" by the highest proportion of respondents (73%) was "Run 10 miles". Other items rated "Not Important" by at least one-half of participants were: "Shovel fresh snow and clear 30 feet of walkway/driveway" (66%); "Row a rowboat" (63%); "Push and move an empty refrigerator" (63%); "Push a car in neutral gear" (61%); "Run five miles" (61%); "Climb 15 flights of stairs (180 steps)" (59%), "Climb 1000 vertical feet on a trail in an hour" (57%), and "Run at a fast pace for two miles" (57%).

##### Respondent feedback

As with floor items, the majority of comments were associated with individual capability or interest in performing the activity or personal reasons for the item's insignificance ("I have towing service...no need to change a tire"). Item-related comments were largely related to requests for greater detail or specification about what was being asked.

#### Final ceiling item pool

The resulting ceiling item pool contained 26 of the original 31 candidate items. We removed the following four items because there was more than 3% data missing responses: "Change a flat tire"; "Shovel fresh snow and clear 30 feet of walkway/driveway"; "Push a car in neutral gear"; and "Climb 1000 vertical feet on a trail in an hour". We also removed the item "Transfer a full load of clothes from a washer to a dryer" because at least 97% of participants had responded: "Without Any Difficulty" indicating that it was actually a floor item. Nineteen (73%) of the items had "Are you able to..." stems. Three had "Does your physical health now limit you..." stems. The remaining items had unique contexts and response option sets, asking about vigorous physical activity, walking and running.

### Study 2: Comparison of item difficulty

#### Respondents

We had a 76% (637/835) ceiling item and a 62% (246/400) floor item response rate (Figure 2). Ceiling item respondents were 69% White, 53% men, averaged 61 years of age, had nearly 17 years of education, and a mean disability score of 0.003. Floor item respondents were mostly White women who averaged 79 years of age with 15 years of education, and a mean disability score of 1.62.

#### Ceiling items

Table 2 displays the 26 final ceiling items (in plain font) compared to the 10 PROMIS PF-10 items (in bolded font) arranged from highest to lowest percent of responses to "With Much Difficulty/Unable to Do". The table shows that the majority of the PROMIS PF-10 items were easier/less difficult to do than the new candidate ceiling items, indicating that the new ceiling items are better at assessing physical function in high functioning participants.

**Table 2:** Comparison of Ceiling item difficulty ratings with PROMIS PF-10 items (bolded font)

	Much difficulty/ unable to do (%)		Much difficulty/ unable to do (%)
■ What do you think is your best time for running 1 mile now?	43	■ Row a rowboat	6
■ Running 10 miles?	41	■ Climb a ladder to trim a tree?	5
■ Running 5 miles?	32	■ How many minutes does it take for you to walk one mile?	5
■ Run at a fast pace for two miles?	27	■ Doing heavy work around the house like scrubbing floors, or lifting or moving heavy furniture?	5
■ Run/jog slowly for two miles?	20	■ **Bending, kneeling, stooping?**	3
■ Over a one-week period, how many times did you do any vigorous physical activity which made you breathe harder or puff and pant?	18	■ Dig a hole in the dirt with a shovel?	3
■ Doing strenuous activities such as backpacking, skiing, playing singles tennis, bicycling or jogging?	12	■ Trim a hedge?	3
■ Climb 15 flights of stairs (60 steps)?	11	■ **Walking more than a mile**?	3
■ Doing eight hours of physical labor?	10	■ Take a 20-minute brisk walk, without stopping to rest?	3
■ In the last week, what do you estimate was the total time that you spent doing vigorous physical activity?	9	■ Move a full garbage/recycle bin?	3
■ Push/move an empty refrigerator?	9	■ Hand wash/wax a car?	2
■ **Doing vigorous activities, such as running, lifting heavy objects, participating in strenuous sports?**	8	■ Do chores such as vacuuming or yard work?	1
■ Climb 10 flights of stairs (40 steps)?	7	■ **Climbing 1 flight of stairs?**	1
		■ **Lifting/carrying groceries?**	1
		■ **Shampoo your hair?**	0
		■ **Wash/dry your body?**	0
		■ **Get on/off toilet?**	0
		■ **Dress yourself, including shoelaces and buttons?**	0

Comparison of item difficulty of the 26 new ceiling items (plain font) with PROMIS PF-10 Items (bolded font) arranged from highest to lowest percent of "With Much Difficulty/Unable to Do" responses.

#### Floor items

Table 3 displays the 30 final floor items (in plain font) compared to the 10 PROMIS PF-10 items (in bolded font) arranged from highest to lowest percent of responses to "With Much Difficulty/Unable to Do". Twenty-six (87%) of the new floor items were rated as being easier/less difficult than the PROMIS PF-10 items, indicating that the new floor items are more applicable than comparison PROMIS PF-10 items to respondents with low physical function abilities.

**Table 3:** Floor items

	Much difficulty/ unable to do (%)		Much difficulty/ unable to do (%)
• **Doing vigorous activities, such as running, lifting heavy objects, participating in strenuous sports?**	88	• Fasten buttons on a shirt or blouse?	25
• **Walking more than a mile?**	81	• Put on your socks?	24
• Walk a block as quickly as you did five years ago?	78	• In the past year, how much weight have you lost unintentionally	24
• Cut your toenails?	70	• In the past year, how many times did you fall?	22
• **Do chores such as vacuuming or yard work?**	66	• Type a sentence on a computer keyboard?	22
• **Bending, kneeling, or stooping?**	65	• Chew and eat your food as quickly as you did five years ago?	20
• Compared to five years ago, is your normal walking speed: Faster, About the same, Slower, Much Slower, Unable to walk)	61	• Wash and dry your body?	18
• **Climbing one flight of stairs?**	56	• Put on your shoes?	17
• Dress and groom yourself as quickly as you did five years ago?	56	• Put on a sweater or t-shirt over your head?	17
• What is the farthest distance you can walk by yourself, without any special equipment or help from others?	55	• Move about your residence?	16
• **Lifting or carrying groceries?**	47	• Get on and off the toilet?	13
• Stand up from an armless straight chair?	40	• Squeeze another person's hand?	13
• Walk up or down inclines?	39	• Use a knife and fork?	13
• Move about in a dark room or hallway without falling?	34	• Move from sitting at the side of the bed to lying down on your back?	11
• Loosen a screw using a manual screwdriver?	31	• Write a simple sentence using a pen or pencil?	11
• Dress yourself in less than 10 minutes?	30	• Get items in and out of a wallet?	10
• Do you feel exhausted?	29	• Pour liquid into a cup?	9
• Move from the street to the sidewalk without a curb cut?	28	• Hold a card or letter in order to read it?	7
• Dress yourself, including shoelaces and doing buttons?	26	• Push the buttons on a television remote control?	7
• Shampoo your hair?	26	• Take a letter out of an envelope?	6
		• Turn pages in a book?	3

Comparison of item difficulty of the 30 final floor items (plain font) with PROMIS PF-10 items (bolded font) arranged from highest to lowest percent of "With Much Difficulty or Unable to Do" responses.

**Figure 4:**
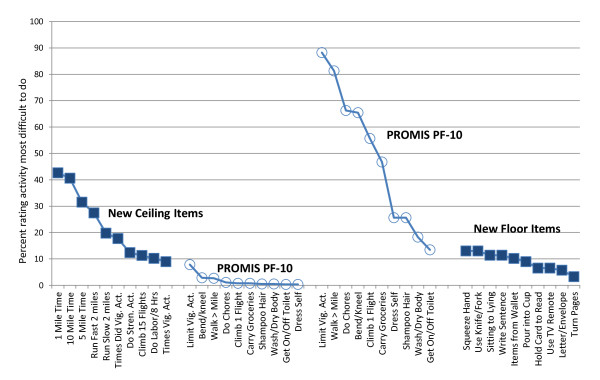
**Comparison of difficulty ratings of new items compared to PROMIS PF 10 items**.

Figure 4 presents the comparison of difficulty ratings for the 10 PROMIS PF-10 items with new ceiling and new floor items. It shows that 10 of the new ceiling items, which had the highest percentage of most difficult to do responses, were rated by high functioning participants (left side of figure) as being 10% to more than 40% more difficult to do than the PROMIS items. The right side of the figure shows that ratings by low functioning participants of 10 new floor items, which had the lowest percentage of most difficult to do responses, were rated as being about 12% to nearly 90% less difficult to do than the PROMIS PF-10 items.

## Discussion

We sought to develop new items to assess the extremes of physical function in order to enable measurement of a more complete range of ability. Essential steps in building an item pool are item identification, construction and evaluation. We employed established PROMIS item development and qualitative evaluation [14] approaches using participants who were representative of several different subpopulations.

These results demonstrate that better items aimed at measuring the extremes of physical function can be developed and assessed using established qualitative evaluation protocols. Our qualitative evaluation of new floor and ceiling items identified items that were clear, important and relevant. Overall, participants found that few of the candidate items were inappropriate and that there were no gaps that needed to be assessed. The qualitative evaluation activities resulted in 26 new ceiling and 30 new floor items that are clear and relevant to both persons with well-above average functioning and persons with below average functioning.

The need for items to extend the ability to assess the floor and the ceiling calls attention to some issues with choosing a metric for floor and ceiling scales. The metric used most often for PROMIS scales is the T-score, where the scale's center is set to the mean of a general population, and each 10 points above or below the mean represents one standard deviation unit [4,14,15]. This scale has consequences which may not be initially obvious. It can only cover five standard deviations above or below the population mean. Often this will not be a problem since it is a broad range, and values at the extremes will not greatly influence results in a general population. However, in a large population there will be people at the tails.

More frequently, there will be a problem because the population being studied is not well represented by the general population mean and distribution. This will usually be the case when a chronic illness, such as rheumatoid arthritis, defines the population, and many people score near the floor. It also happens when a study population consists of persons who are young and fit and have scores approaching the ceiling. A suggestion hence may be to consider a T-score where the population mean and standard deviation is described by the study population itself. This may result in IRT calibrations being different from the general population and perhaps the need for population-specific calibrations. These issues require further investigation, which we will be addressing in the ongoing validation study of the floor and ceiling items presented here.

Moreover, studies such as these raise practical issues. It is necessary to identify appropriate items, which must be either very easy or very hard, yet they must be within the frame of reference of the study population. Items that address mountain climbing or running a 100-mile race are not within the typical frame of reference for many people. This issue may be ameliorated by using different types of response options, such as time to complete an activity, or comparing abilities in an earlier time frame, such as over a five-year span. Maintaining a frame of reference is also necessary to avoid items from appearing inappropriate or even offensive. To test new floor and ceiling items it is also necessary to have suitable subjects for item evaluation, and most subject selection methods will apply to several rather different sets of altered abilities, such as spinal cord injuries and dementia. These challenges may be overcome but require careful study design and execution.

There are of course limitations to our studies. These findings may not necessarily apply to those less educated or other seniors who reside independently in their own homes. Individuals at the floor with different disease pathologies might demonstrate differential item functioning (DIF). We were able to conduct only one focus group for only the floor items. However, we have generally found that cognitive surveys using defined populations of 100 or more individuals are preferable to focus groups in order to detect unusual problems that occur at a low level, such as at 5% or 10% of participants.

## Conclusions

The development of items to fill in measurement gaps at the floor and the ceiling is an important contribution to the measurement of PROs. These new floor and ceiling items will particularly help to improve instrument performance in populations concentrated at one or the other extreme end of functioning. A careful approach to a disease-specific set of floor items has recently been developed by other PROMIS-related groups. This work is intended to be specific to outcome assessment of spinal cord injury patients [16,17].

These new Physical Function items are presently undergoing IRT calibration and validation in a one-year longitudinal study, which will enable their use in computerized adaptive testing (CAT) applications. CAT applications are well-suited for diverse populations. They dynamically select the best items for each individual sequentially, based upon their responses to earlier items. CAT will provide additional flexibility for administration and valid measurement across the large spectrum of physical functioning, reduce questionnaire burden, help to insure administration to appropriate individuals, and will represent an important advancement in physical function assessment across the spectrum of functional ability. Reduction in floor and ceiling effects will also be essential in domains other than physical function, allowing more rigorous estimation of abilities at the lower and upper ranges of a population and study of diverse disease populations.

Legacy instruments, such as the HAQ, leave 50% of subjects with scores of zero in broad populations inaccessible, and many more not accessible at the floor. There are two major consequences: first, that measurement precision is much less than desirable; and second, that the ability to accurately measure health improvements of the public is implicitly limited.

## Abbreviations

ARAMIS: Arthritis: Rheumatism: and Aging Medical Information System; CAT: Computerized Adaptive Testing; HAQ: Health Assessment Questionnaire Disability Index; IRT: Item Response Theory; PF: Physical Function; PRO: Patient Reported Outcome; PROMIS: Patient Reported Outcomes Measurement Information System; PROMIS PF-10: PROMIS Physical Function 10-item Short Form; PROMIS PF-20: PROMIS Physical Function 20-item Short Form; SF-36: Stanford Form-36 item physical function form.

## Competing Interests

The authors declare that they have no competing interests.

## Authors' contributions

BB contributed to concept and design, analysis and data interpretation, statistical analyses, and manuscript drafting and review. JFF contributed to concept and design, analysis and data interpretation, and manuscript drafting and review. BL conducted statistical analyses and manuscript review. YNH contributed to participant recruitment and manuscript review. EK contributed to concept and design, data interpretation and manuscript review. All authors read and approved the final manuscript.
